# Gender-specific economic interventions for women’s mental health and well-being in South Asia: a systematic review

**DOI:** 10.1007/s00127-026-03074-8

**Published:** 2026-03-05

**Authors:** Jaya Katiyar, Roxanne Keynejad

**Affiliations:** 1 Centre for Global Mental Health, Health Service and Population Research Department, Institute of Psychiatry, Psychology & Neuroscience, King’s College London, London, UK; 2 Centre for Global Mental Health, London School of Hygiene Tropical Medicine, London, UK; 3 Addictions and Related Research Group, Sangath, Goa India; 4 King’s Women’s Mental Health, Health Service and Population Research Department, Institute of Psychiatry, Psychology and Neuroscience, King’s College London, London, UK

**Keywords:** Economic interventions, Women’s mental health, Gender and mental health, Common mental disorders, South Asia

## Abstract

**Background:**

The prevalence of common mental disorders (CMDs) in South Asia is higher among women than men. Reasons include lower socioeconomic status and greater exposure to social, economic and environmental disadvantage. Economic determinants are important, modifiable risk factors for CMDs.

**Aims:**

This systematic review aimed to evaluate the evidence for impacts of economic interventions on women’s well-being and CMD outcomes in South Asia.

**Method:**

We searched for peer-reviewed quantitative research studies in eight databases (3ie, EconLit, Embase Classic, Embase, Global Health, J-PAL, Medline, APA PsychInfo, Scopus) and studies included in relevant systematic reviews of economic interventions which measured CMDs and well-beingamong female participants in South Asia. We assessed the risk of bias of included studies using the Joanna Briggs Institute checklist. Following data extraction, we synthesised our findings through narrative synthesis.

**Results:**

We identified eight eligible studies evaluating five types of economic intervention: microfinance, employment, conditional cash transfers, self-help groups (SHGs) and economic empowerment programmes. Studies were conducted in Afghanistan (*n* = 1), Bangladesh (*n* = 2) and India (*n* = 5). Approximately 60% of studies reported statistically significant associations between receipt of economic interventions and improved CMD symptoms or well-being among female participants. Risk of bias was moderate and study designs were heterogeneous. Attention to gendered risks of economic interventions in countries with strong patriarchal norms was limited.

**Conclusion:**

The limited published evidence suggests that economic interventions can have positive impacts on CMDs and well-being among women in South Asia. However, methodological limitations and lack of geographical representation mean that further research is needed to evaluate the risks and benefits of economic interventions for diverse women in South Asia.

**Supplementary Information:**

The online version contains supplementary material available at https://doi.org/10.1007/s00127-026-03074-8.

## Introduction

Globally, mental health conditions contribute approximately 20% to the global disease burden and are the second largest contributor to years lived with disability [1, 2]. Common mental disorders (CMDs), particularly depression and anxiety disorders, account for 8% of years lived with disability [3], although this burden is unequally distributed across regions, socioeconomic status and gender [4, 5]. CMD prevalence has been increasing worldwide, especially in low and middle-income countries (LMICs), where they contribute up to 16.6% of the total burden of disease [6, 7].

In the South Asian region (defined by the World Bank [8] as Afghanistan, Bangladesh, Bhutan, India, Maldives, Nepal, Pakistan, and Sri Lanka), the prevalence of CMDs has risen to 14.2%: somewhat higher than the globally reported prevalence of 13.4% [6]. A meta-analysis reported a pooled adult prevalence of 16.0% for depression, 12.0% for anxiety disorders, and 14.0% for depression and anxiety combined in South Asia [9]. Factors influencing CMD prevalence include demographic and lifestyle changes and inadequate mental health service provision [6, 9]. The gap between demand and available treatment is exacerbated by inadequate provision of services addressing the social determinants of mental health [10, 11].

Social determinants of mental health comprise demographic, economic, neighbourhood, environmental and socio-cultural factors, including poverty [11], which influence the prevalence and severity of mental health conditions across the life course [4, 10, 12]. International agencies such as the Organisation for Economic Co-operation and Development (OECD) and World Economic Forum have prioritised improving mental health in their policy objectives [13]. CMDs, particularly, are associated with economic factors. People living with relatively low income are up to three times more likely to experience CMDs than people with high incomes, especially in LMICs with stark income inequities [3, 14].

In the South Asian region, economic inequities are compounded by gender inequities, increasing women’s risk of poor mental health [15, 16]. Compared to men, women in South Asia have less access to financial resources, lower incomes, and experience more violence and discrimination, including financial abuse [5, 17]. These forms of disadvantage are compounded by women’s limited access to education and employment opportunities, which further constrain their economic autonomy [18–20] Consequently, women are more likely to have low socioeconomic status than men within the same household, and therefore suffer the effects of poverty disproportionately [3, 15, 17]. Women in South Asia are thus at increased risk of CMDs relative to men, especially depression and anxiety disorders [1, 2, 21]. The risk of CMDs is heightened by women’s low utilisation of health care services, driven by barriers such as stigma towards ill-health in women, especially mental ill-health [19].

Relationships between poverty, gender and CMDs suggest that interventions addressing economic determinants could impact women’s mental health in South Asia. Woman-centred economic interventions such as conditional and unconditional cash transfer programmes, employment schemes, self-help groups (SHGs) and microfinance are prominent in the region [22, 23]. These approaches aim to promote gender-specific dimensions of economic empowerment, such as female decision-making power, financial agency, economic independence and social connections [20, 22]. Potential mechanisms by which economic interventions might improve CMDs include reduced food insecurity, debt, financial strain, and deprivation, acquisition of income-generating skills, and enhanced employment opportunities [2, 4]. However, evidence for the mental health impact of economic interventions in South Asia is limited. A recent umbrella review identified 24 systematic reviews of economic interventions’ impact on mental health but the majority were conducted in high-income countries (HICs), evaluated only certain types of intervention or measured household or age-specific population outcomes [2]. Three systematic reviews identified 12, 17 and 45 studies respectively, which evaluated the mental health impacts of cash transfer programmes in LMICs [13, 24, 25]. However, none focused specifically on women or South Asia. All three reviews reported findings aligned with those from HICs, suggesting that income improvements are associated with improved mental health.

With less than 10% of global mental health research attributed to South Asian countries [26], little is known about the impact of economic interventions on CMDs and mental well-being among women in the region. This study is the first to synthesise the evidence for the impact of economic interventions on women’s mental health in South Asia. Our objectives were:

- To summarise available evidence on economic interventions delivered to women in South Asia, which measured CMD symptoms or well-being outcomes.
- To identify any associations between economic intervention delivery and CMD symptoms or well-being among women living in South Asia.
- To identify gaps in the peer-reviewed literature requiring further investigation.

## Methods

This systematic review followed Preferred Reporting Items for Systematic reviews and Meta-Analyses (PRISMA) guidelines [27] (supplementary file 1). The protocol was prospectively registered on the PROSPERO international prospective register of systematic reviews (registration number: CRD42024577928).

### Search strategy

We conducted a systematic search of the following databases via the OVID online platform: Embase Classic, Embase (1947 to 2024 July 04), APA PsychInfo (1806 to July Week 1 2024), EconLit (1886 to June 27, 2024), Global Health (1910 to 2024 Week 26), Medline (R) ALL (1946 to July 04, 2024), and Scopus (1979–2024). We hand-searched for ‘grey’ literature in the 3ie Repository for Impact Evaluation and Abdul Latif Jameel Poverty Action Lab (J-PAL). We screened bibliographies and lists of studies included in relevant systematic reviews identified during the search.

We used the Boolean operator “OR” to find articles using multiple synonymous search terms and “AND” to combine different concepts. We used truncation and wildcards to account for spelling and terminology variations. The full search strategy is provided in supplementary file 2.

### Study selection

Studies retrieved from each database were transferred to Rayyan [28], a free artificial intelligence-powered online tool, to aid the screening process and remove duplicates.

### Eligibility criteria

We defined the eligibility criteria using the Population, Intervention, Comparison, Outcome, Setting (PICOS) framework. The population of interest was female participants aged 18 years or older, either as the primary sample or as a sub-group, for whom data were disaggregated. Any intervention type focused on mitigating economic hardship was eligible, such as cash transfers (conditional or unconditional), SHGs or employment programmes. Studies combining non-economic (e.g. psychological) interventions with economic interventions were eligible for inclusion if data could be disaggregated for each component. This approach ensured that only effects directly attributable to economic interventions were included in our evidence synthesis. Due to the anticipated low number of eligible studies, we did not exclude studies based on control or comparison groups. Eligible studies were required to measure at least one CMD symptom or well-being with standardised, validated instruments. The study setting was required to be at least one country within the World Bank-defined South Asian region, namely Afghanistan, Bangladesh, Bhutan, India, Maldives, Nepal, Pakistan, Sri Lanka. We did not exclude studies based on language or publication year. All intervention evaluation designs were eligible: experimental studies, quasi-experimental studies, observational studies and mixed methods studies with an evaluation component.

### Data extraction and synthesis

We prepared a data extraction template in Microsoft Excel 2021 for Windows, informed by previous relevant systematic reviews. We extracted data on study author, year of publication, aim, population and sample characteristics, study design, outcome descriptions, outcome measures, intervention type(s), study duration and key findings). Following extraction, we synthesised the data in a narrative format, focused on evidence for the impact of economic interventions on CMDs and the well-being of women in South Asia and developed a summary of findings table. A planned meta-analysis was not possible because of the heterogeneity of interventions, study designs and risk of bias in included studies. Data extraction began on 10/08/24.

### Risk of bias assessment

We assessed included studies using Joanna Briggs Institute (JBI) critical appraisal tools [29–31]. We adopted a scale-based approach to assess the risk of bias [32] (supplementary file 3).

## Results

### Study selection

**Fig. 1:**
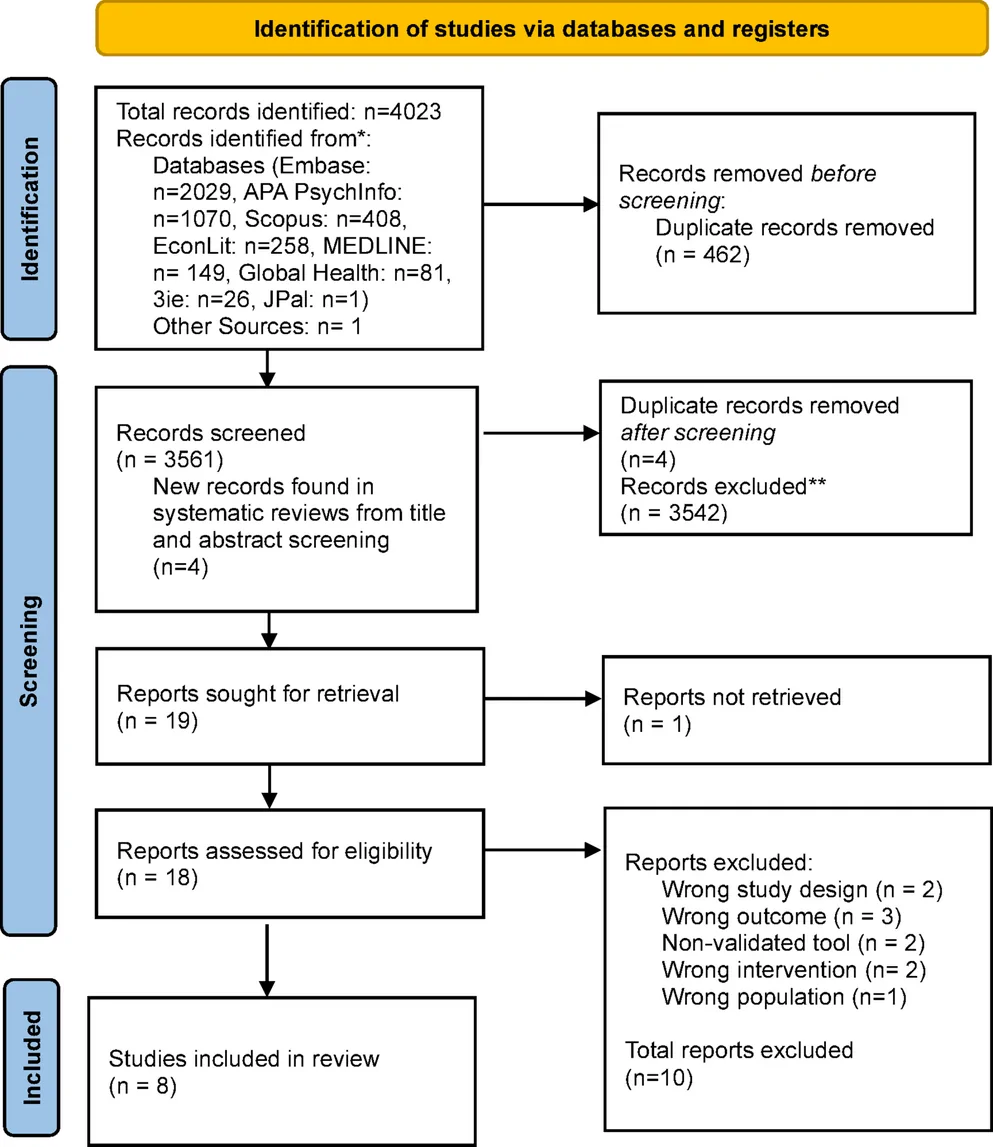
PRISMA flowchart

We retrieved a total of 4023 articles from our searches of eight databases and the bibliographies of relevant systematic reviews. After exporting to Rayyan, we electronically excluded 462 duplicate articles. We selected 19 records for full-text screening, of which we were unable to obtain one article. At full text screening, we excluded 10 studies. These comprised three studies which reported ineligible outcomes [33–35], twoqualitative non-evaluation study designs [36, 37], two non-economic interventions [38, 39] two studies which used non-validated outcome measures [40, 41] and one study of an ineligible population [42]. Eight studies were eligible to be included in this systematic review, of which one study used an ICD-10 diagnostic criteria-based questionnaire rather than a standardised instrument [43]. The study selection process is reported in the PRISMA flowchart in Fig. 1.

### Study characteristics

Table 1 summarises the characteristics of included studies. All studies were published between 2010 and 2021 in English. Only three out of eight South Asian countries were represented: Afghanistan (*n* = 1) [44], Bangladesh (*n* = 2) [45, 46] and India (*n* = 5) [43, 47–50]. Of the studies conducted in India, two studies reported research conducted with the same community in South India, at different time points [47, 48]. Most studies enrolled adult females aged 18 to 49 years. Only two studies enrolled women aged 50 years and older. Total sample sizes varied from 64 to 2476 women; only two studies provided a priori power calculations for their sample sizes [44, 50] and one provided *post-hoc* power calculations [49]. Three studies were secondary data analyses of larger surveys, and one study was a sub-study of a parent study [43, 45, 46, 50].

Study designs included one randomised controlled trial (RCT) [44], three quasi-experimental studies [43, 45, 49], and four cross-sectional studies [46–48, 50]. Five out of eight included studies compared outcomes of the intervention to a control or comparison group. Only the RCT measured CMD symptoms or well-being at both baseline and endline.

### Intervention characteristics

Five types of economic intervention were evaluated by included studies. One study from India [47] and two studies from Bangladesh [45, 46] assessed microfinance programmes, comprising loans, micro-savings and skills training, implemented by non-governmental organisations. Two studies, both from India, evaluated women-only SHGs [48, 50], of which one assessed the SHG component of a larger microcredit programme, which was also included in this systematic review [47]. One study [43] from India evaluated an employment programme. Another study from India [49] focused on conditional cash transfers contingent on giving birth at a health facility. The RCT evaluated a multi-component programme aiming to empower Afghan women economically and socially through human rights, vocational and microfinance training [44].

**Table 1:** Characteristics of included studies

Study References	Country	Participants	Study Design	Data Source	Sample Size	Age	Intervention/Programme	Implementation Organisation	Outcome
**Kayser et al.(2010)**	India	Widowed or ‘abandoned’ women	Cross-sectional	Data collected for the study	109	Mean age: 45 years Age range: 22-75 years	Microfinance: loans to re-establish livelihoods, self-help groups (SHGs), skills training, legal counselling and social support. The primary focus was the microcredit component: loans and investments	Non-governmental organisation (NGO)	Psychological effects: perceived self-value and benefits from participating
**Newransky**,** Kayser & Lombe (2014)**	India	Widowed or ‘abandoned’ women	Cross-sectional	Data collected for the study	64	Mean age: 43 years Age range: 28 to 70 years	SHGs: each SHG had two leaders who helped women to manage savings and book keeping, disseminate information and solve problems within the group. All members were required to attend training sessions comprising motivational speeches, discussions, and marches for community awareness raising.	NGO	Self-efficacy:ability to solve one's own problems Overall well-being: degree of worry about not having necessities to live and function in society
**Powell-Jackson et al. (2016)**	India	Women who gave birth in the last two years	Quasi-experimental design with a control group: women who gave birth in a health facility but who did not receive the cash transfer	Data collected for the study	1695: Intervention group: 1589 Control group: 106	15-49 years	Conditional cash transfer ‘Janani Suraksha Yojana’: one-off cash transfers to women who give birth in a health facility	Government	Maternal depression(Kessler Psychological Distress Scale) Emotional well-being: happy/ worried/ depressed
**Tsaneva & Balakrishnan (2019)**	India	Women from rural households	Quasi-experimental design with a control group: women from urban households	*WHO study on global AGEing and adult health (SAGE)* dataset	2476	18 years and above	Employment programme: the National Rural Employment Guarantee Scheme (NREGS) provided 100 days of low-skilled, paid work at minimum wage to rural households in projects like road construction, irrigation and sanitation	Government	Depression (ICD-10 item questions)
**Scott et al.(2020)**	India	Women with children aged between six and 24 months old	Cross-sectional	Sub study of *Women Improving Nutrition through Group-based Strategies (WINGS)*	1644: Intervention group: approximately 707 Control group: approximately 937 (manually calculated)	Mean age: 26 years Age range: 18-46 years	SHGs: focused on agricultural livelihood strengthening activities	NGO	CMD (20-item self-reporting questionnaire (SRQ))
**Jalal**,** Frongillo & Warren (2015)**	Bangladesh	Women living in ultra-poor households	Quasi-experimental design with a control group ofhouseholds that were part of the programme but no longer fit the inclusion criteria because they are economically better off	Data collected from a larger survey in 2006	209: Intervention group: 110 Control group: 99	Mean ages: Intervention group: 29.25 years Control group: 27.26 years	Microfinance: Challenging the Frontiers of Poverty Reduction-Targeting the Ultra Poor (CFPR-TUP): the poverty alleviation programme provided income-earning assets, subsistence allowance, and basic entrepreneurial skills training. Income generating activities were supervised for 10 months, after which women graduated into the Bangladesh Rural Advancement Committeemicro-credit programme.	NGO	DistressSubjective well-being (Positive Affect and Negative Affect Schedule)
**Yount et al.(2021)**	Bangladesh	Married women	Cross-sectional design with a control group: women who had never participated in micro-finance	Sub study of *Intimate Partner Coercion and Implications for Women’s Health and Well-Being*	921: Intervention group: 497 Control group: 434	15 to 49 years	Microfinance: services included micro-credits, micro-savings, micro-insurance and cash transfers. Participants engaged in kin-based, village-based or occupation-based savings groups or microfinance organizations	NGO	Depressive symptoms (Center for Epidemiologic Studies Depression (CES-D)-10 scale)
**Gibbs et al.(2020)**	Afghanistan	Women from conflict-affected communities with signs of economic vulnerability (extreme poverty, unemployment, high risk or unsafe occupations) and with no severe mental health problems or disability	Randomised controlled trial (RCT) control group: women who did not participate in the programme	Data collected for the study	1461: Intervention group: 747 at baseline, 673 at endline Control group: 714 at baseline, 537 at endline	18-49 years	Economic and social empowerment programme: 1. Rights-based training delivered to groups of 25 women in community-based training centres with numeracy, business skills, social empowerment, hands-on training in a chosen vocational skill. 2. Monthly stipend of US $10 3. Introduction to formal and informal saving mechanisms (SHGs and microfinance) 4. Referrals to health, legal and financial services 5. Connections to other women	NGO	Depressive symptoms (20 item Center for Epidemiologic Studies Depression Scale) Life satisfaction

**Fig. 2:**
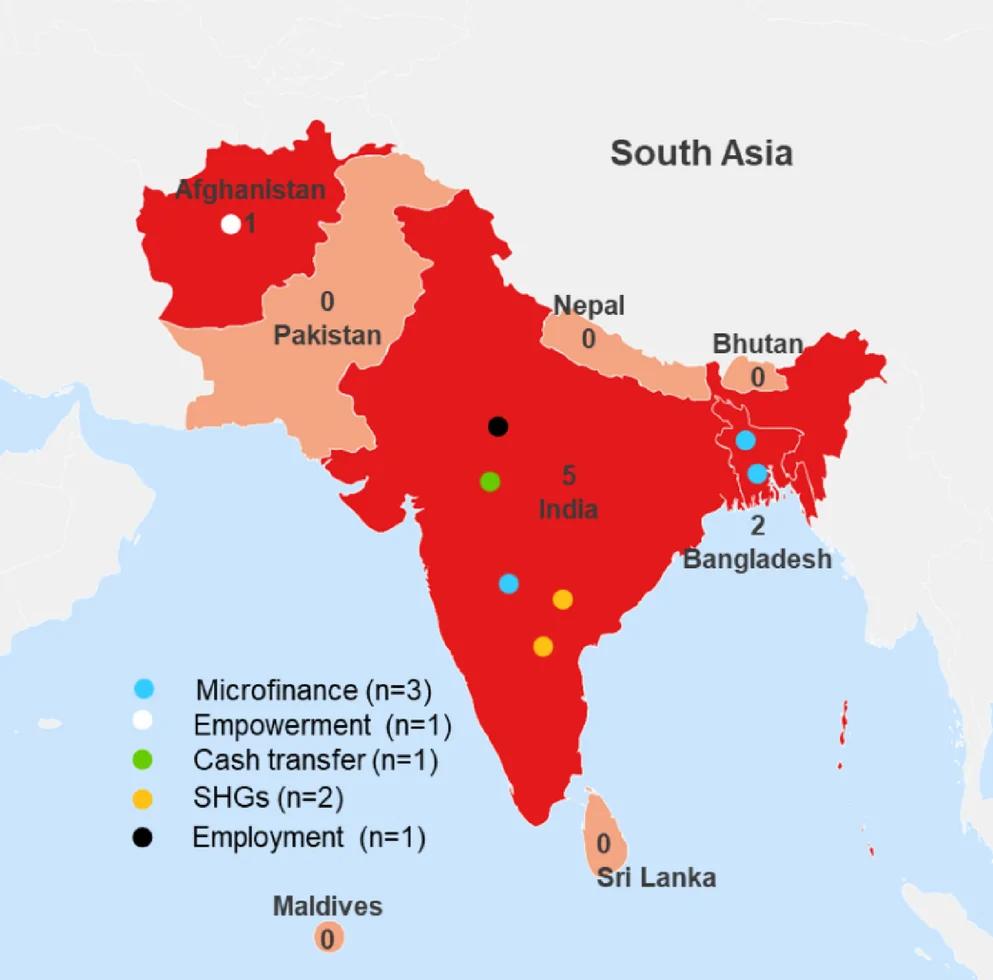
Regional variation of included studies

Figure 2 shows the distribution of identified economic interventions assessed in South Asia.

### Outcome measurement

Five studies [43, 46–48, 50] reported either CMD symptoms or well-being as outcomes and three studies measured both [44, 45, 49].

### Common mental disorders

Depressive symptoms were the most-often measured CMD outcome [43–46, 49] using heterogeneous instruments. Studies used versions of the Centre for Epidemiologic Studies Depression scale (CES-D; *n* = 2) [44, 46], Kessler Psychological Distress Scale (K-10; *n* = 1) [49], 20 item Self Report Questionnaire (SRQ; *n* = 2) [45, 50] and a questionnaire based on the International Classification of Diseases (ICD-10) diagnostic criteria for depression (*n* = 1) [43].

### Well-being

Five studies reported measures of well-being [44, 45, 47–49], conceptualised as emotional well-being, subjective well-being, overall well-being, psychological effects, and life satisfaction.

Only two studies measured well-being using externally developed tools [44, 45]: the Positive Affect Schedule and Negative Affect Schedule and Satisfaction with Life Scale. Two studies developed their own scales and provided Cronbach’s alpha scores for reliability [47, 48]. One study integrated well-being-related questions into their ICD-10 diagnostic criteria-based questionnaire [43] and another created a five-point Likert scale to assess well-being [49].

### Risk of bias assessment

Risk of bias assessments of each study are summarised in Table 2. Three studies—two cross-sectional [46, 50] and one RCT [44]—had low risk of bias and the remaining five—two cross-sectional [47, 48] and three quasi-experimental [43, 45, 49]—had moderate risk of bias. Identification of and controlling for confounders were a common source of bias. In cross-sectional studies, the reliability and validity of outcome measures were at risk of bias for two studies [47, 48]. No quasi-experimental studies measured outcomes at baseline or longer-term follow-up post-intervention [43, 45, 49]. For more details on risk of bias scoring see supplementary file 3.

### Impact of economic interventions on outcomes

The key findings of included studies are summarised in Table 2. Five out of eight studies (63%) reported a statistically significant association between economic intervention receipt and at least one mental health or well-being outcome. The other three studies identified non-significant improvements in the hypothesised direction, suggesting that they may have been inadequately powered to detect clinical efficacy [43, 44, 49].

### Common mental disorder symptoms

Of the six studies measuring CMD symptoms, three (50%) reported significant improvement following economic intervention. Two were quasi-experimental studies evaluating a microfinance programme and a cash transfer programme. Of these, one study reported that compared to the control arm, the microfinance programme was associated with a 74% reduction in distress in the intervention arm, with improved food security the most significant mediator [45]. The second study reported an 8.5% reduction in maternal depressive symptoms (adjusted mean difference: -0.09 (95% CI -0.16 to -0.019), *p* = 0.013) and a 36% reduction in moderate depressive symptoms (adjusted odds ratio: 0.64 (95% CI 0.47 to 0.89), *p* = 0.008) [49]. The third study identifying a significant improvement was a cross-sectional study, which found that 44% of women reported no CMD symptoms after participating in a SHG (adjusted odds ratio: 0.73 (95% CI 0.56 to 0.96), *p* < 0.05) [50]. The remaining three (50%) studies measuring CMD symptoms —one RCT of an economic empowerment programme and two cross-sectional studies of microfinance and employment programmes —found no statistically significant effects of economic intervention on women's mental health. The RCT found non-significant effect sizes (adjusted odds ratio: -0.35 (95% CI -1.19 to 0.48), *p* = 0.40) [44]. The cross-sectional study of a microfinance programme reported average treatment effects (propensity scores) of 0.052 (95% CI -0.045 to 0.150), SE 0.05, *p* = 0.29 [46]. The cross-sectional study of an employment programme [43] reported an adjusted probability of depression score of 0.056 (SE = 0.0377, *p* > 0.10), which remained non-significant after sensitivity analysis (adjusted probability of depression score= -0.047; SE = 0.0345, *p* > 0.10).

### Well-being

Of the five studies measuring well-being, two (40%) reported a statistically significant impact of economic interventions on well-being. Of these, the quasi-experimental study of a microfinance programme reported a 30% improvement in well-being with food security as the most significant mediator [45]. The cross-sectional study of SHGs observed that self-efficacy associated with SHGs was a strong predictor of overall well-being: unstandardised mean difference *b* = 2.68, SE = 0.87, t = 3.07, *p* < 0.01 [48]. Two studies (40%) found no statistically significant effects of economic interventions on well-being. The RCT [44] of an empowerment programme observed non-significant effect sizes for life satisfaction (adjusted odds ratio: 0.09 (95% CI -0.12 to 0.29), *p* = 0.39). The quasi-experimental study of conditional cash transfers [49] reported non-significant z-scores for well-being (‘happy’: z-score = 0.12 (95% CI -0.094 to 0.33), *p* = 0.27; ‘worried’: z-score= -0.14 (95% CI -0.35 to 0.065), *p* = 0.17). The cross-sectional study [47] found no statistically significant associations between microfinance and ‘psychological effects’ (unstandardised mean difference b = 2.38, *p* = 0.02) but did identify a significant association between microfinance-embedded SHG participation and psychological effects (unstandardised mean difference b= -0.09, SE = 0.04; t= -2.49, *p* < 0.05).

**Table 2:** Summary of findings

Study References	Outcome	Instrument of Measurement	Statistical Results	Key Findings	Risk of Bias
Kayser et al. (2010)	Psychological effects of self-value and benefits from participating in the programme	Psychological Effects Index	*Adjusted unstandardized mean difference*: Participating in the micro-credit programme b = 2.38, *p* = 0.02. Duration of SHG participation b= -0.09, SE 0.04; t=-2.49, *p* < 0.05	The loan size and type of investment had non-significant impacts on psychological effects. There was a significant association between SHG membership and psychological effects; recently formed groups were associated with greater well-being outcomes.	Moderate
Newransky, Kayser & Lombe (2014)	Self-efficacy: Frequency of participation. Number of visits by facilitator	3 point Likert Scale	*Adjusted odds ratio*: Frequency of participation= 4.10, *p* ≤ 0.05. Number of visits by facilitator= 4.67, *p* ≤ 0.05	Participating in SHGs and regular guidance by facilitators was significantly associated with women’s self-efficacy. Improved self-efficacy was associated with high levels of overall well-being.	Moderate
	Overall well-being	4 point Likert Scale	*Adjusted unstandardised mean difference (self-efficacy as predictor)*: 2.68, SE 0.87, t = 3.07, *p* < 0.01		
Powell-Jackson et al. (2016)	Maternal depression	Kessler Psychological Distress Scale (K-10)	*Continuous variable: Adjusted mean difference*: -0.09 (95% CI -0.16 to -0.019), *p* = 0.013 *Categorical variable: Adjusted odds ratio*: Severe depression= 0.37 (95% CI 0.12 to 1.08), *p* = 0.07 Moderate depression= 0.64 (95% CI 0.47 to 0.89), *p* = 0.008	Participating in the programme was associated with reduced maternal depressive symptoms compared towomene in the control group. This association remained following sensitivity analyses. For emotional well-being, only reduced depressive symptoms were significantly associated with programme participation. Receipt of cash transfers was associated with lower risk of delivery care-related debt (a financial stressor and potential risk factor for CMDs).	Moderate
	Emotional well-being: ‘happy’, ‘worried’, ‘depressed’	5 point Likert Scale (z-score)	*z-score* happy = 0.11 (95% CI -0.097 to 0.32) worried= -0.12 (95% CI -0.32 to 0.086) depressed= -0.22 (95% CI -0.41 to -0.02)		
Tsaneva & Balakrishnan (2019)	Depression	ICD-10 diagnostic criteria-based questionnaire with follow-up questions on psychological well-being, future thinking, agency and control	*Adjusted probability of depression*: 0.056, SE = 0.037, *p* > 0.10 *Post-sensitivity analyses Adjusted probability of depression* 0.047, SE = 0.034, *p* > 0.10	Women who received the employment programme had a lower probability of depression compared to women who did not receive the intervention, but this association was not statistically significant.	Moderate
Scott et al. (2020)	CMDs	20-Item Self Reporting Questionnaire (SRQ)	*Adjusted odds ratio* 0.73 (95%CI 0.56 to 0.96), *p* < 0.05	44% of SHG members reported no CMD symptoms. There was a significant association between SHG membership and lowerCMD symptoms.	Low
Jalal, Frongillo & Warren (2015)	Distress	WHO Self-Reporting Questionnaire (SRQ)	*Effect of programme on distress* -1.68 (directly); -4.39 (through stressors) & -0.46 (through perceived economic status at midline)	74.2% of the total programme effect on *distress* was indirect, via impacts on domestic violence, food insecurity, perceived economic and social constraints. The remaining 25.8% was the programme’s direct effect. 30% of the programme’s effect on *well-being* was indirect and 70% was direct. For both outcomes, food insecurity was the most important mediator as it mediated more that 50% of the total indirect effect of the programme.	Moderate
	Subjective well-being	Positive Affect and Negative Affect Schedule	*Effect of programme on well-being* 8.42 (directly); 0.13 (indirectly)		
Yount et al. (2021)	Depressive symptoms	Center for Epidemiologic Studies Depression (CES-D)-10 scale	*Average treatment effect (propensity score)* 0.052 (95% CI -0.045 to 0.150), SE = 0.05, *p* = 0.295	Microfinance participation was not significantly associated with depressive symptoms.	Low
Gibbs et al. (2020)	Depressive symptoms	20 item Center for Epidemiologic Studies Depression (CES-D)	*Adjusted odds ratio* -0.35 (95% CI -1.19 to 0.48), *p* = 0.406	The intervention was not significantly associated with improved depression or life satisfaction although the measured change was in the hypothesised direction.	Low
	Life satisfaction	Satisfaction with Life Scale	*Adjusted odds ratio* 0.09 (95% CI -0.12 to 0.29), *p* = 0.395		

## Discussion

This systematic review synthesised available evidence on the impact of economic interventions on the mental health of women living in the South Asian region. Eight studies conducted in India, Bangladesh, and Afghanistan, evaluating five distinct economic interventions were eligible for inclusion. Except one RCT, studies employed observational or quasi-experimental designs. These studies measured CMD symptoms and well-being using heterogeneous instruments; depressive symptoms were the most commonly reported mental health outcome.

More than 60% of included studies reported a statistically significant impact of economic programmes on CMD symptoms and well-being of participating women. The direction of the relationship between participation and mental health was consistently found to be in the hypothesised direction, raising the possibility that some designs were under-powered to detect efficacy. Compared to other economic interventions included in this systematic review, SHGs were consistently reported to have a positive impact on CMD outcomes and well-being.

Two included studies reported improvements in economic well-being. One of these reported increased decision-making power, greater mobility, and higher overall saving through an economic and social empowerment programme [44]. The second study identified food security as important mediator of economic well-being following a microfinance programme [45]. Both studies reported positive changes in mental health outcomes, suggesting associations between economic well-being and women’s mental health in these South Asian contexts. The absence of a negative association between economic programmes and mental health outcomes indicates that no adverse effects of economic interventions on women’s mental health were reported in the peer-reviewed literature. Our review therefore suggests the potential for some economic interventions to improve mental health outcomes for adult women in specific contexts. This is in line with recent systematic reviews of economic interventions’ impact on the mental health of mixed populations [2, 13, 24, 25, 51].

We identified broadly positive published reports of the overall impact and mechanisms through which economic interventions may improve women’s mental health in South Asia. By increasing economic mobility, income-generating skills and opportunities, and providing social and economic support through loans, cash transfers and SHGs, economic interventions may plausibly improve the mental health of women. However, with 40% of studies reporting non-significant effects, predominantly moderate risk of bias and the likelihood of publication bias, these findings must be appraised in the context of included studies’ methodological limitations. Additionally, none of the included studies measured or reported the longer-term sustainability of the observed effects of economic interventions on women’s mental health outcomes.

The mixed nature of reported effects may arise in part from heterogeneity of included interventions (the quantity and forms of financial assistance, inclusion of livelihood strengthening activities, frequency of training sessions). The diversity of intervention components limited the comparability of programmes. Considerable variation in study designs is likely to have contributed to our mixed findings. Dominant non-randomised quasi-experimental or cross-sectional designs were susceptible to selection bias and confounding. However, designing, implementing and evaluating interventions on social determinants of mental health is challenging and the field benefits from reporting of real-world socio-ecological characteristics which influence interventions’ efficacy, in practice [2, 4]. Observational and quasi-experimental designs can be especially useful for evaluating large-scale programmes, as was the case for several economic interventions included in this review. These studies provide evidence on real-world outcomes and experiences , essential to develop a contextualised evidence base [52].

Heterogeneity of CMD symptom and well-being measures may also have contributed to mixed findings. A variety of scales was used, with several studies employing, non-validated scales. Few studies conducted CMD symptom screening or applied clinical cut-offs, given the non-mental health focus of most interventions. Other than the RCT, no study measured outcomes at baseline or longer-term post-intervention follow-up. This may have overestimated the impact of these interventions, in the absence of baseline measures and control groups, or underestimated the impact, as mental health outcomes may be better captured by longitudinal evaluations [50]. Only three studies provided sample size calculations, increasing the likelihood that studies were under-powered to detect statistically significant effects.

An additional consideration is that of selection bias. Women’s participation in social and economic activities outside home may be under the jurisdiction of their households, such as partners or family elders [53, 54]. The samples of included studies may therefore lack representation from those women prevented from participating by their families and so exclude an important proportion of the affected population.

Another consideration is the potential unintended consequences of complex economic intervention programmes. One study highlighted that stress induced by participating in microfinance programmes can affect well-being [46]. Similarly, failure to address systemic issues can limit the impact of economic interventions. For example, some studies evaluated economic programmes delivered to vulnerable women but did not address factors such as caste-based discrimination, and lasting trauma from past conflicts [44, 47–49]. A study of conditional cash transfers noted that their impact was diminished by a poorly functioning public health system which delayed payment disbursement [49]. Only two studies reported intimate partner or domestic violence exposure among participants [45,46]. The RCT conducted in post-conflict Afghanistan noted that the integrated empowerment programme did not improve depression because it could not address women’s profoundly disempowered status on a socio-political level [44]. The studies we identified did not acknowledge, measure, or mitigate for gendered risks to participants, such as potential exclusion, hostility or discrimination provoked by perceived threats to patriarchal norms [20]. Global evidence reports that participation in microfinance initiatives is associated with increased employment of women but is also associated with household instability arising from conflicts with traditional gender roles [55, 56]. One study reported that although the conditional cash transfer programme included in this review was associated with improved obstetric outcomes, maternal mortality remained high, suggesting that female-specific discrimination may have shifted from the ante-natal to the post-natal period [57]. Lack of attention to these unintended consequences is a concern in settings of high gender-based violence prevalence. Given the potential for complex repercussions on established social processes, future economic intervention studies should integrate safety considerations into every stage of their design and implementation.

SHGs had a consistent positive impact on CMD symptoms and well-being. Beyond economic empowerment, SHGs empower women by strengthening peer and sociopolitical networks [48, 58]. Evidence suggests that women participating in SHGs are more confident, politically active, and aware of their entitlements [22, 58]. Research is needed to investigate the mechanisms by which such complex, gender-sensitive interventions can improve women’s mental health and gender equity. Integrating economic interventions with other interventions targeting domains like health, social protection and equity might mitigate gendered risks and improve mental health outcomes. Of the eight included studies, around 60% were from India, reflecting regional inequality in research capacity and limiting the applicability of findings to the wider South Asian region and LMICs more broadly. Research investment is urgently needed, which rigorously evaluates interventions to improve women’s mental health in under-represented countries in South Asia. Many such interventions are already being implemented by voluntary sector organisations but their evaluations, where conducted, are not widely accessible, and often lack baseline measures and control groups.

### Strengths and limitations

This systematic review provides a comprehensive overview of the known mental health impacts of published economic interventions for women in low and middle-income country settings of South Asia. By including studies measuring both CMDs and well-being, we synthesised evidence for a holistic conceptualisation of women’s mental health [3, 4, 19]. We employed broad inclusion criteria and considered a range of economic intervention models, in any language. Limitations included single reviewer screening and searching of only one grey literature database, potentially excluding relevant articles, including from countries not represented in this review.

## Conclusions

This systematic review contributes to the growing literature on the intersection between financial empowerment, gender and mental health. Our findings align with evidence from both high-income and low-and-middle income settings from male and female populations [51]. Given mixed findings from included studies, this review suggests potential mental health benefits of economic interventions as social protection strategies, which require further investigation. While no adverse effects on mental health outcomes were reported, potential risks and harms, including gender-based violence, financial risks, and programme implementation issues require careful consideration by future, rigorously conducted evaluations. Further research is needed to equip policy-makers with a better understanding of associations between economic well-being and women’s mental health. Realist methods can be used to explore the mechanisms through which economic well-being can improve women's mental health through hypothesised mechanisms such as empowerment. Wider impacts of economic interventions need to be explored by evaluating multiple outcomes including mental health, economic security and empowerment.

Included studies evaluated other outcomes such as physical health, social capital, household health status and intimate partner violence exposure, generally reporting improvements. The potential multidomain impacts of economic interventions imply that they can be effective strategies to supportpopulations facing multiple, intersecting life adversities. Studies exploring the impacts of complex, multicomponent graduation-style or empowerment programmes are needed. Future prospective research should evaluate the long-term effects of economic interventions in comparison with control arms, taking baseline and follow-up measures. Evidence regarding the feasibility, acceptability and cost-effectiveness of economic interventions is also required to support future scale up. Studies conducted across the South Asian region are needed to ensure that the emerging evidence base is context-appropriate, culturally sensitive and responsive to policy-makers’ evidence needs. 

## Supplementary Information

Below is the link to the electronic supplementary material.

Supplementary Material 1

## Data Availability

No datasets were generated or analysed during the current study.
