# Supplementary material for: Gender-specific economic interventions for women’s mental health and well-being in South Asia: a systematic review
Source: Soc Psychiatry Psychiatr Epidemiol. 2026 Mar 5;61(12):2493–505. doi: 10.1007/s00127-026-03074-8 (PMC13727933; doi:10.1007/s00127-026-03074-8)
Supplement: Supplementary file 1 [file 127_2026_3074_MOESM1_ESM.docx]

Supplementary 1

**PRISMA 2020 Main Checklist**

|  |  | Reporting Item | Page Number |
| --- | --- | --- | --- |
| **Title** |  |  |  |
| Title | [#1](https://www.goodreports.org/reporting-checklists/prisma/info/#1) | Identify the report as a systematic review | 1 |
| **Abstract** |  |  |  |
| Abstract | [#2](https://www.goodreports.org/reporting-checklists/prisma/info/#2) | Report an abstract addressing each item in the PRISMA 2020 for Abstracts checklist | 2 |
| **Introduction** |  |  |  |
| Background/rationale | [#3](https://www.goodreports.org/reporting-checklists/prisma/info/#3) | Describe the rationale for the review in the context of existing knowledge | 3-4 |
| Objectives | [#4](https://www.goodreports.org/reporting-checklists/prisma/info/#4) | Provide an explicit statement of the objective(s) or question(s) the review addresses | 4 |
| **Methods** |  |  |  |
| Eligibility criteria | [#5](https://www.goodreports.org/reporting-checklists/prisma/info/#5) | Specify the inclusion and exclusion criteria for the review and how studies were grouped for the syntheses | 5-6 |
| Information sources | [#6](https://www.goodreports.org/reporting-checklists/prisma/info/#6) | Specify all databases, registers, websites, organisations, reference lists, and other sources searched or consulted to identify studies. Specify the date when each source was last searched or consulted | 5 |
| Search strategy | [#7](https://www.goodreports.org/reporting-checklists/prisma/info/#7) | Present the full search strategies for all databases, registers, and websites, including any filters and limits used | 5, supplementary 2 |
| Selection process | [#8](https://www.goodreports.org/reporting-checklists/prisma/info/#8) | Specify the methods used to decide whether a study met the inclusion criteria of the review, including how many reviewers screened each record and each report retrieved, whether they worked independently, and, if applicable, details of automation tools used in the process | 5-6 |
| Data collection process | [#9](https://www.goodreports.org/reporting-checklists/prisma/info/#9) | Specify the methods used to collect data from reports, including how many reviewers collected data from each report, whether they worked independently, any processes for obtaining or confirming data from study investigators, and, if applicable, details of automation tools used in the process | 6 |
| Data items | [#10a](https://www.goodreports.org/reporting-checklists/prisma/info/#10a) | List and define all outcomes for which data were sought. Specify whether all results that were compatible with each outcome domain in each study were sought (for example, for all measures, time points, analyses), and, if not, the methods used to decide which results to collect | 5 |
| Study risk of bias assessment | [#11](https://www.goodreports.org/reporting-checklists/prisma/info/#11) | Specify the methods used to assess risk of bias in the included studies, including details of the tool(s) used, how many reviewers assessed each study and whether they worked independently, and, if applicable, details of automation tools used in the process | 6 |
| Effect measures | [#12](https://www.goodreports.org/reporting-checklists/prisma/info/#12) | Specify for each outcome the effect measure(s) (such as risk ratio, mean difference) used in the synthesis or presentation of results | NA |
| Synthesis methods | [#13a](https://www.goodreports.org/reporting-checklists/prisma/info/#13a) | Describe the processes used to decide which studies were eligible for each synthesis (such as tabulating the study intervention characteristics and comparing against the planned groups for each synthesis (item #5)) | 6 |
| Synthesis methods | [#13b](https://www.goodreports.org/reporting-checklists/prisma/info/#13b) | Describe any methods required to prepare the data for presentation or synthesis, such as handling of missing summary statistics or data conversions | NA |
| Synthesis methods | [#13c](https://www.goodreports.org/reporting-checklists/prisma/info/#13c) | Describe any methods used to tabulate or visually display results of individual studies and syntheses | 6 |
| Synthesis methods | [#13d](https://www.goodreports.org/reporting-checklists/prisma/info/#13d) | Describe any methods used to synthesise results and provide a rationale for the choice(s). If meta-analysis was performed, describe the model(s), method(s) to identify the presence and extent of statistical heterogeneity, and software package(s) used | 6 |
| Synthesis methods | [#13e](https://www.goodreports.org/reporting-checklists/prisma/info/#13e) | Describe any methods used to explore possible causes of heterogeneity among study results (such as subgroup analysis, meta-regression) | NA |
| Synthesis methods | [#13f](https://www.goodreports.org/reporting-checklists/prisma/info/#13f) | Describe any sensitivity analyses conducted to assess robustness of the synthesised results | NA |
| Reporting bias assessment | [#14](https://www.goodreports.org/reporting-checklists/prisma/info/#14) | Describe any methods used to assess risk of bias due to missing results in a synthesis (arising from reporting biases) | NA |
| Certainty assessment | [#15](https://www.goodreports.org/reporting-checklists/prisma/info/#15) | Describe any methods used to assess certainty (or confidence) in the body of evidence for an outcome | NA |
| Data items | [#10b](https://www.goodreports.org/reporting-checklists/prisma/info/#10b) | List and define all other variables for which data were sought (such as participant and intervention characteristics, funding sources). Describe any assumptions made about any missing or unclear information | 5 |
| **Results** |  |  |  |
| Study selection | [#16a](https://www.goodreports.org/reporting-checklists/prisma/info/#16a) | Describe the results of the search and selection process, from the number of records identified in the search to the number of studies included in the review, ideally using a flow diagram (http://www.prisma-statement.org/PRISMAStatement/FlowDiagram) | 7 |
| Study selection | [#16b](https://www.goodreports.org/reporting-checklists/prisma/info/#16b) | Cite studies that might appear to meet the inclusion criteria, but which were excluded, and explain why they were excluded | 7 |
| Study characteristics | [#17](https://www.goodreports.org/reporting-checklists/prisma/info/#17) | Cite each included study and present its characteristics | 8, Table 1 |
| Risk of bias in studies | [#18](https://www.goodreports.org/reporting-checklists/prisma/info/#18) | Present assessments of risk of bias for each included study | 14, Table 2 |
| Results of individual studies | [#19](https://www.goodreports.org/reporting-checklists/prisma/info/#19) | For all outcomes, present for each study (a) summary statistics for each group (where appropriate) and (b) an effect estimate and its precision (such as confidence/credible interval), ideally using structured tables or plots | Table 2 |
| Results of syntheses | [#20a](https://www.goodreports.org/reporting-checklists/prisma/info/#20a) | For each synthesis, briefly summarise the characteristics and risk of bias among contributing studies | 14-15, Table 2 |
| Results of syntheses | [#20b](https://www.goodreports.org/reporting-checklists/prisma/info/#20b) | Present results of all statistical syntheses conducted. If meta-analysis was done, present for each the summary estimate and its precision (such as confidence/credible interval) and measures of statistical heterogeneity. If comparing groups, describe the direction of the effect | NA |
| Results of syntheses | [#20c](https://www.goodreports.org/reporting-checklists/prisma/info/#20c) | Present results of all investigations of possible causes of heterogeneity among study results | NA |
| Results of syntheses | [#20d](https://www.goodreports.org/reporting-checklists/prisma/info/#20d) | Present results of all sensitivity analyses conducted to assess the robustness of the synthesised results | NA |
| Risk of reporting biases in syntheses | [#21](https://www.goodreports.org/reporting-checklists/prisma/info/#21) | Present assessments of risk of bias due to missing results (arising from reporting biases) for each synthesis assessed | NA |
| Certainty of evidence | [#22](https://www.goodreports.org/reporting-checklists/prisma/info/#22) | Present assessments of certainty (or confidence) in the body of evidence for each outcome assessed | NA |
| **Discussion** |  |  |  |
| Results in context | [#23a](https://www.goodreports.org/reporting-checklists/prisma/info/#23a) | Provide a general interpretation of the results in the context of other evidence | 19-21 |
| Limitations of included studies | [#23b](https://www.goodreports.org/reporting-checklists/prisma/info/#23b) | Discuss any limitations of the evidence included in the review | 19-21 |
| Limitations of the review methods | [#23c](https://www.goodreports.org/reporting-checklists/prisma/info/#23c) | Discuss any limitations of the review processes used | 21 |
| Implications | [#23d](https://www.goodreports.org/reporting-checklists/prisma/info/#23d) | Discuss implications of the results for practice, policy, and future research | 21 |
| **Other information** |  |  |  |
| Registration and protocol | [#24a](https://www.goodreports.org/reporting-checklists/prisma/info/#24a) | Provide registration information for the review, including register name and registration number, or state that the review was not registered | 5 |
| Registration and protocol | [#24b](https://www.goodreports.org/reporting-checklists/prisma/info/#24b) | Indicate where the review protocol can be accessed, or state that a protocol was not prepared | 5 |
| Registration and protocol | [#24c](https://www.goodreports.org/reporting-checklists/prisma/info/#24c) | Describe and explain any amendments to information provided at registration or in the protocol | NA |
| Support | [#25](https://www.goodreports.org/reporting-checklists/prisma/info/#25) | Describe sources of financial or non-financial support for the review, and the role of the funders or sponsors in the review | NA |
| Competing interests | [#26](https://www.goodreports.org/reporting-checklists/prisma/info/#26) | Declare any competing interests of review authors | NA |
| Availability of data, code, and other materials | [#27](https://www.goodreports.org/reporting-checklists/prisma/info/#27) | Report which of the following are publicly available and where they can be found: template data collection forms; data extracted from included studies; data used for all analyses; analytic code; any other materials used in the review | NA |

Notes:

- 7: 5, supplementary 2
- 18: 14, Table 2
- 20a: 14-15, Table 2 The PRISMA checklist is distributed under the terms of the Creative Commons Attribution License CC-BY. This checklist was completed on 29. December 2024 using <https://www.goodreports.org/>, a tool made by the [EQUATOR Network](https://www.equator-network.org) in collaboration with [Penelope.ai](https://www.penelope.ai)

Supplementary 2

**Search Strategy**

| Embase Classic + Embase (1947 to 2024 July 04) | 1 Afghanistan/ 7593  2 Afghanistan.ti,ab. 8871  3 Nepal/ 16937  4 Nepal.ti,ab. 17003  5 or/1-4 [LOW INCOME COUNTRIES IN SOUTH ASIA] 30468  6 Bangladesh/ 23294  7 Bangladesh.ti,ab. 24332  8 Bhutan/ 1285  9 Bhutan.ti,ab. 1319  10 exp India/ 205979  11 India.ti,ab. 202922  12 exp Pakistan/ 40578  13 Pakistan.ti,ab. 39617  14 or/6-13 [LOWER MIDDLE INCOME COUNTRIES IN SOUTH ASIA] 340585  15 maldives/ 500  16 Maldives.ti,ab. 600  17 Sri Lanka/ 11732  18 (Sri Lanka or Ceylon).ti,ab. 11977  19 or/15-18 [UPPER MIDDLE INCOME COUNTRIES IN SOUTH ASIA] 14924  20 1 or 2 or 3 or 4 or 5 or 6 or 7 or 8 or 9 or 10 or 11 or 12 or 13 or 14 or 15 or 16 or 17 or 18 or 19 378021  21 female/ 12686309  22 wom#n.mp. 2102833  23 21 or 22 12947181  24 20 and 23 176833  25 exp mental health/ 268274  26 exp "Depression, Anxiety and Stress Scale"/ or exp Self-rating Depression Scale/ or exp atypical depression/ or exp Hamilton Depression Rating Scale/ or exp long term depression/ or exp antenatal depression/ or exp adolescent depression/ or exp depression assessment/ or exp major depression/ or exp Montgomery Asberg Depression Rating Scale/ or exp "mixed anxiety and depression"/ or exp Center for Epidemiological Studies Depression Scale/ or exp perinatal depression/ or exp Major Depression Inventory/ or exp Beck Depression Inventory/ or exp chronic depression/ or exp "Depression, Anxiety and Stress Scale-21"/ or exp depression inventory/ or exp bipolar depression/ or exp minor depression/ or exp "Depression, Anxiety and Stress Scale-42"/ or exp depression/ 726370  27 exp short health anxiety inventory/ or exp Generalized Anxiety Disorder-2/ or exp "Depression, Anxiety and Stress Scale"/ or exp anxiety assessment/ or exp State Trait Anxiety Inventory/ or exp anxiety/ or exp "mixed anxiety and depression"/ or exp Generalized Anxiety Disorder Scale/ or exp illness anxiety disorder/ or exp "anxiety/defense test battery"/ or exp anxiety disorder/ or exp Generalized Anxiety Disorder-7/ or exp Hamilton Anxiety Scale/ or exp Zung Self Rating Anxiety Scale/ or exp "Depression, Anxiety and Stress Scale-21"/ or exp Anxiety Sensitivity Index/ or exp "mood and anxiety symptom questionnaire"/ or exp generalized anxiety disorder/ or exp Self-rating Anxiety Scale/ or exp "Depression, Anxiety and Stress Scale-42"/ or exp Beck Anxiety Inventory/ 640233  28 exp posttraumatic stress disorder checklist/ or exp emotional stress/ or exp acute stress/ or exp family stress/ or exp mental stress/ or exp stress assessment/ or exp life stress/ or exp Perceived Stress Scale/ or exp acute stress disorder/ or exp maternal stress/ or exp "Depression, Anxiety and Stress Scale-21"/ or exp job stress/ or exp behavioral stress/ or exp "Depression, Anxiety and Stress Scale"/ or exp posttraumatic stress disorder/ or exp financial stress/ 314378  29 exp wellbeing/ 134333  30 exp emotion/ or exp wellbeing/ or exp female/ or exp emotional well-being/ or exp mental health/ or exp psychological aspect/ 13588964  31 exp mental health/ or exp psychological aspect/ or exp psychological well-being/ or exp female/ or exp wellbeing/ 13232374  32 psychosocial stress*.mp. [mp=title, abstract, heading word, drug trade name, original title, device manufacturer, drug manufacturer, device trade name, keyword heading word, floating subheading word, candidate term word] 10368  33 common mental disorder*.mp. [mp=title, abstract, heading word, drug trade name, original title, device manufacturer, drug manufacturer, device trade name, keyword heading word, floating subheading word, candidate term word] 4833  34 mental ill*.mp. [mp=title, abstract, heading word, drug trade name, original title, device manufacturer, drug manufacturer, device trade name, keyword heading word, floating subheading word, candidate term word] 61162  35 psychological well-being.mp. [mp=title, abstract, heading word, drug trade name, original title, device manufacturer, drug manufacturer, device trade name, keyword heading word, floating subheading word, candidate term word] 43574  36 emotional well-being.mp. [mp=title, abstract, heading word, drug trade name, original title, device manufacturer, drug manufacturer, device trade name, keyword heading word, floating subheading word, candidate term word] 12469  37 25 or 26 or 27 or 28 or 29 or 30 or 31 or 32 or 33 or 34 or 35 or 36 13931708  38 24 and 37 174152  39 economic intervention*.mp. [mp=title, abstract, heading word, drug trade name, original title, device manufacturer, drug manufacturer, device trade name, keyword heading word, floating subheading word, candidate term word] 232  40 cash transfer*.mp. [mp=title, abstract, heading word, drug trade name, original title, device manufacturer, drug manufacturer, device trade name, keyword heading word, floating subheading word, candidate term word] 1309  41 poverty alleviation*.mp. [mp=title, abstract, heading word, drug trade name, original title, device manufacturer, drug manufacturer, device trade name, keyword heading word, floating subheading word, candidate term word] 894  42 poverty reduction.mp. [mp=title, abstract, heading word, drug trade name, original title, device manufacturer, drug manufacturer, device trade name, keyword heading word, floating subheading word, candidate term word] 837  43 social transfer.mp. [mp=title, abstract, heading word, drug trade name, original title, device manufacturer, drug manufacturer, device trade name, keyword heading word, floating subheading word, candidate term word] 173  44 social assistance.mp. [mp=title, abstract, heading word, drug trade name, original title, device manufacturer, drug manufacturer, device trade name, keyword heading word, floating subheading word, candidate term word] 1527  45 social welfare.mp. [mp=title, abstract, heading word, drug trade name, original title, device manufacturer, drug manufacturer, device trade name, keyword heading word, floating subheading word, candidate term word] 26489  46 social income.mp. [mp=title, abstract, heading word, drug trade name, original title, device manufacturer, drug manufacturer, device trade name, keyword heading word, floating subheading word, candidate term word] 24  47 income support.mp. [mp=title, abstract, heading word, drug trade name, original title, device manufacturer, drug manufacturer, device trade name, keyword heading word, floating subheading word, candidate term word] 451  48 livelihood*.mp. [mp=title, abstract, heading word, drug trade name, original title, device manufacturer, drug manufacturer, device trade name, keyword heading word, floating subheading word, candidate term word] 6997  49 basic grant.mp. [mp=title, abstract, heading word, drug trade name, original title, device manufacturer, drug manufacturer, device trade name, keyword heading word, floating subheading word, candidate term word] 9  50 insurance*.mp. [mp=title, abstract, heading word, drug trade name, original title, device manufacturer, drug manufacturer, device trade name, keyword heading word, floating subheading word, candidate term word] 304236  51 loan*.mp. [mp=title, abstract, heading word, drug trade name, original title, device manufacturer, drug manufacturer, device trade name, keyword heading word, floating subheading word, candidate term word] 3847  52 39 or 40 or 41 or 42 or 43 or 44 or 45 or 46 or 47 or 48 or 49 or 50 or 51 343596  53 38 and 52 2029 |
| --- | --- |
| APA PsychInfo 1806 to July Week 1 2024 | 1. female*.mp.  2. wom#n.mp. [mp=title, abstract, heading word, table of contents, key concepts, original title, tests & measures, mesh word]  3. 1 or 2  4. exp Mental Health/  5. depression*.mp.  6. exp Generalized Anxiety Disorder/ or exp Anxiety Disorders/ or exp Anxiety/ or exp Anxiety Screening/ or exp State Trait Anxiety Inventory/  7. exp Acute Stress/ or exp Chronic Stress/ or exp Posttraumatic Stress Disorder/ or exp Stress/ or exp Acute Stress Disorder/ or exp Posttraumatic Stress/ or exp Psychological Stress/  8. exp Well Being/  9. emotional well-being.mp.  10. psychological well-being.mp.  11. psychosocial stress*.mp. [mp=title, abstract, heading word, table of contents, key concepts, original title, tests & measures, mesh word]  12. exp Anxiety/ or exp Anxiety Disorders/ or exp Major Depression/ or common mental disorder*.mp.  13. mental ill*.mp.  14. 4 or 5 or 6 or 7 or 8 or 9 or 10 or 11 or 12 or 13  15. 3 and 14  16. exp Poverty/ or exp Mental Health/ or exp Economics/ or exp Intervention/ or exp Socioeconomic Status/ or exp Empowerment/ or exp Health Promotion/  17. cash transfer*.mp. [mp=title, abstract, heading word, table of contents, key concepts, original title, tests & measures, mesh word]  18. poverty alleviation.mp. [mp=title, abstract, heading word, table of contents, key concepts, original title, tests & measures, mesh word]  19. poverty reduction.mp. [mp=title, abstract, heading word, table of contents, key concepts, original title, tests & measures, mesh word]  20. social transfer.mp. [mp=title, abstract, heading word, table of contents, key concepts, original title, tests & measures, mesh word]  21. social assistance.mp. [mp=title, abstract, heading word, table of contents, key concepts, original title, tests & measures, mesh word]  22. social welfare.mp. [mp=title, abstract, heading word, table of contents, key concepts, original title, tests & measures, mesh word]  23. social income.mp. [mp=title, abstract, heading word, table of contents, key concepts, original title, tests & measures, mesh word]  24. income support.mp. [mp=title, abstract, heading word, table of contents, key concepts, original title, tests & measures, mesh word]  25. livelihood*.mp. [mp=title, abstract, heading word, table of contents, key concepts, original title, tests & measures, mesh word]  26. basic grant.mp. [mp=title, abstract, heading word, table of contents, key concepts, original title, tests & measures, mesh word]  27. insurance*.mp. [mp=title, abstract, heading word, table of contents, key concepts, original title, tests & measures, mesh word]  28. loan*.mp. [mp=title, abstract, heading word, table of contents, key concepts, original title, tests & measures, mesh word]  29. 16 or 17 or 18 or 19 or 20 or 21 or 22 or 23 or 24 or 25 or 26 or 27 or 28  30. 15 and 29  31. south asia.mp. [mp=title, abstract, heading word, table of contents, key concepts, original title, tests & measures, mesh word]  32. india.mp. [mp=title, abstract, heading word, table of contents, key concepts, original title, tests & measures, mesh word]  33. maldives.mp. [mp=title, abstract, heading word, table of contents, key concepts, original title, tests & measures, mesh word]  34. pakistan.mp. [mp=title, abstract, heading word, table of contents, key concepts, original title, tests & measures, mesh word]  35. nepal.mp. [mp=title, abstract, heading word, table of contents, key concepts, original title, tests & measures, mesh word]  36. bhutan.mp. [mp=title, abstract, heading word, table of contents, key concepts, original title, tests & measures, mesh word]  37. bangladesh.mp. [mp=title, abstract, heading word, table of contents, key concepts, original title, tests & measures, mesh word]  38. sri lanka.mp. [mp=title, abstract, heading word, table of contents, key concepts, original title, tests & measures, mesh word]  39. afghanistan.mp. [mp=title, abstract, heading word, table of contents, key concepts, original title, tests & measures, mesh word]  40. global south.mp. [mp=title, abstract, heading word, table of contents, key concepts, original title, tests & measures, mesh word]  41. 31 or 32 or 33 or 34 or 35 or 36 or 37 or 38 or 39 or 40  42. 30 and 41 |
| Econlit 1886 to June 27 2024 | 1. female*.mp. [mp=heading words, abstract, title, country as subject]  2. wom#n.mp. [mp=heading words, abstract, title, country as subject]  3. 1 or 2  4. mental health.mp. [mp=heading words, abstract, title, country as subject]  5. depress*.mp. [mp=heading words, abstract, title, country as subject]  6. anxiety.mp. [mp=heading words, abstract, title, country as subject]  7. well-being.mp. [mp=heading words, abstract, title, country as subject]  8. psychological well-being.mp. [mp=heading words, abstract, title, country as subject]  9. emotional well-being.mp. [mp=heading words, abstract, title, country as subject]  10. psychosocial stress*.mp. [mp=heading words, abstract, title, country as subject]  11. stress*.mp. [mp=heading words, abstract, title, country as subject]  12. common mental disorder*.mp. [mp=heading words, abstract, title, country as subject]  13. mental ill*.mp. [mp=heading words, abstract, title, country as subject]  14. 4 or 5 or 6 or 7 or 8 or 9 or 10 or 11 or 12 or 13  15. 3 and 14  16. economic intervention*.mp. [mp=heading words, abstract, title, country as subject]  17. cash transfer*.mp. [mp=heading words, abstract, title, country as subject]  18. poverty alleviation.mp. [mp=heading words, abstract, title, country as subject]  19. poverty reduction.mp. [mp=heading words, abstract, title, country as subject]  20. social transfer.mp. [mp=heading words, abstract, title, country as subject]  21. social assistance.mp. [mp=heading words, abstract, title, country as subject]  22. social welfare.mp. [mp=heading words, abstract, title, country as subject]  23. social income.mp. [mp=heading words, abstract, title, country as subject]  24. income support.mp. [mp=heading words, abstract, title, country as subject]  25. livelihood*.mp. [mp=heading words, abstract, title, country as subject]  26. basic grant*.mp. [mp=heading words, abstract, title, country as subject]  27. insurance*.mp. [mp=heading words, abstract, title, country as subject]  28. loan*.mp. [mp=heading words, abstract, title, country as subject]  29. 16 or 17 or 18 or 19 or 20 or 21 or 22 or 23 or 24 or 25 or 26 or 27 or 28  30. 15 and 29  31. south asia.mp. [mp=heading words, abstract, title, country as subject]  32. global south.mp. [mp=heading words, abstract, title, country as subject]  33. india.mp. [mp=heading words, abstract, title, country as subject]  34. maldives.mp. [mp=heading words, abstract, title, country as subject]  35. pakistan.mp. [mp=heading words, abstract, title, country as subject]  36. nepal.mp. [mp=heading words, abstract, title, country as subject]  37. bhutan.mp. [mp=heading words, abstract, title, country as subject]  38. sri lanka.mp. [mp=heading words, abstract, title, country as subject]  39. afghanistan.mp. [mp=heading words, abstract, title, country as subject]  40. 31 or 32 or 33 or 34 or 35 or 36 or 37 or 38 or 39  41. 30 and 40 |
| Global Health 1910 to 2024 Week 26 | 1. exp females/  2. (women or girls).sh.  3. 1 or 2  4. economics.sh. or exp mental health/ or mental disorders.sh.  5. depression/  6. anxiety.mp. [mp=title, book title, abstract, original title, name of substance word, subject heading word, floating sub-heading word, keyword heading word, organism supplementary concept word, protocol supplementary concept word, rare disease supplementary concept word, unique identifier, synonyms, population supplementary concept word, anatomy supplementary concept word]  7. exp mental stress/ or exp stress/  8. well-being.mp.  9. emotional well-being.mp. [mp=title, book title, abstract, original title, name of substance word, subject heading word, floating sub-heading word, keyword heading word, organism supplementary concept word, protocol supplementary concept word, rare disease supplementary concept word, unique identifier, synonyms, population supplementary concept word, anatomy supplementary concept word]  10. psychological well-being.mp. [mp=title, book title, abstract, original title, name of substance word, subject heading word, floating sub-heading word, keyword heading word, organism supplementary concept word, protocol supplementary concept word, rare disease supplementary concept word, unique identifier, synonyms, population supplementary concept word, anatomy supplementary concept word]  11. psychosocial stress*.mp. [mp=title, book title, abstract, original title, name of substance word, subject heading word, floating sub-heading word, keyword heading word, organism supplementary concept word, protocol supplementary concept word, rare disease supplementary concept word, unique identifier, synonyms, population supplementary concept word, anatomy supplementary concept word]  12. common mental disorder*.mp. [mp=title, book title, abstract, original title, name of substance word, subject heading word, floating sub-heading word, keyword heading word, organism supplementary concept word, protocol supplementary concept word, rare disease supplementary concept word, unique identifier, synonyms, population supplementary concept word, anatomy supplementary concept word]  13. mental ill*.mp. [mp=title, book title, abstract, original title, name of substance word, subject heading word, floating sub-heading word, keyword heading word, organism supplementary concept word, protocol supplementary concept word, rare disease supplementary concept word, unique identifier, synonyms, population supplementary concept word, anatomy supplementary concept word]  14. 4 or 5 or 6 or 7 or 8 or 9 or 10 or 11 or 12 or 13  15. 3 and 14  16. economic intervention*.mp. [mp=title, book title, abstract, original title, name of substance word, subject heading word, floating sub-heading word, keyword heading word, organism supplementary concept word, protocol supplementary concept word, rare disease supplementary concept word, unique identifier, synonyms, population supplementary concept word, anatomy supplementary concept word]  17. cash transfer*.mp. [mp=title, book title, abstract, original title, name of substance word, subject heading word, floating sub-heading word, keyword heading word, organism supplementary concept word, protocol supplementary concept word, rare disease supplementary concept word, unique identifier, synonyms, population supplementary concept word, anatomy supplementary concept word]  18. poverty alleviation*.mp. [mp=title, book title, abstract, original title, name of substance word, subject heading word, floating sub-heading word, keyword heading word, organism supplementary concept word, protocol supplementary concept word, rare disease supplementary concept word, unique identifier, synonyms, population supplementary concept word, anatomy supplementary concept word]  19. poverty reduction.mp. [mp=title, book title, abstract, original title, name of substance word, subject heading word, floating sub-heading word, keyword heading word, organism supplementary concept word, protocol supplementary concept word, rare disease supplementary concept word, unique identifier, synonyms, population supplementary concept word, anatomy supplementary concept word]  20. social transfer.mp. [mp=title, book title, abstract, original title, name of substance word, subject heading word, floating sub-heading word, keyword heading word, organism supplementary concept word, protocol supplementary concept word, rare disease supplementary concept word, unique identifier, synonyms, population supplementary concept word, anatomy supplementary concept word]  21. social assistance.mp. [mp=title, book title, abstract, original title, name of substance word, subject heading word, floating sub-heading word, keyword heading word, organism supplementary concept word, protocol supplementary concept word, rare disease supplementary concept word, unique identifier, synonyms, population supplementary concept word, anatomy supplementary concept word]  22. social welfare.mp. [mp=title, book title, abstract, original title, name of substance word, subject heading word, floating sub-heading word, keyword heading word, organism supplementary concept word, protocol supplementary concept word, rare disease supplementary concept word, unique identifier, synonyms, population supplementary concept word, anatomy supplementary concept word]  23. social income.mp. [mp=title, book title, abstract, original title, name of substance word, subject heading word, floating sub-heading word, keyword heading word, organism supplementary concept word, protocol supplementary concept word, rare disease supplementary concept word, unique identifier, synonyms, population supplementary concept word, anatomy supplementary concept word]  24. income support.mp. [mp=title, book title, abstract, original title, name of substance word, subject heading word, floating sub-heading word, keyword heading word, organism supplementary concept word, protocol supplementary concept word, rare disease supplementary concept word, unique identifier, synonyms, population supplementary concept word, anatomy supplementary concept word]  25. livelihood*.mp. [mp=title, book title, abstract, original title, name of substance word, subject heading word, floating sub-heading word, keyword heading word, organism supplementary concept word, protocol supplementary concept word, rare disease supplementary concept word, unique identifier, synonyms, population supplementary concept word, anatomy supplementary concept word]  26. basic grant*.mp. [mp=title, book title, abstract, original title, name of substance word, subject heading word, floating sub-heading word, keyword heading word, organism supplementary concept word, protocol supplementary concept word, rare disease supplementary concept word, unique identifier, synonyms, population supplementary concept word, anatomy supplementary concept word]  27. insurance*.mp. [mp=title, book title, abstract, original title, name of substance word, subject heading word, floating sub-heading word, keyword heading word, organism supplementary concept word, protocol supplementary concept word, rare disease supplementary concept word, unique identifier, synonyms, population supplementary concept word, anatomy supplementary concept word]  28. loan*.mp. [mp=title, book title, abstract, original title, name of substance word, subject heading word, floating sub-heading word, keyword heading word, organism supplementary concept word, protocol supplementary concept word, rare disease supplementary concept word, unique identifier, synonyms, population supplementary concept word, anatomy supplementary concept word]  29. 16 or 17 or 18 or 19 or 20 or 21 or 22 or 23 or 24 or 25 or 26 or 27 or 28  30. 15 and 29  31. south asia.mp. [mp=title, book title, abstract, original title, name of substance word, subject heading word, floating sub-heading word, keyword heading word, organism supplementary concept word, protocol supplementary concept word, rare disease supplementary concept word, unique identifier, synonyms, population supplementary concept word, anatomy supplementary concept word]  32. global south.mp. [mp=title, book title, abstract, original title, name of substance word, subject heading word, floating sub-heading word, keyword heading word, organism supplementary concept word, protocol supplementary concept word, rare disease supplementary concept word, unique identifier, synonyms, population supplementary concept word, anatomy supplementary concept word]  33. india.mp. [mp=title, book title, abstract, original title, name of substance word, subject heading word, floating sub-heading word, keyword heading word, organism supplementary concept word, protocol supplementary concept word, rare disease supplementary concept word, unique identifier, synonyms, population supplementary concept word, anatomy supplementary concept word]  34. maldives.mp. [mp=title, book title, abstract, original title, name of substance word, subject heading word, floating sub-heading word, keyword heading word, organism supplementary concept word, protocol supplementary concept word, rare disease supplementary concept word, unique identifier, synonyms, population supplementary concept word, anatomy supplementary concept word]  35. pakistan.mp. [mp=title, book title, abstract, original title, name of substance word, subject heading word, floating sub-heading word, keyword heading word, organism supplementary concept word, protocol supplementary concept word, rare disease supplementary concept word, unique identifier, synonyms, population supplementary concept word, anatomy supplementary concept word]  36. nepal.mp. [mp=title, book title, abstract, original title, name of substance word, subject heading word, floating sub-heading word, keyword heading word, organism supplementary concept word, protocol supplementary concept word, rare disease supplementary concept word, unique identifier, synonyms, population supplementary concept word, anatomy supplementary concept word]  37. bhutan.mp. [mp=title, book title, abstract, original title, name of substance word, subject heading word, floating sub-heading word, keyword heading word, organism supplementary concept word, protocol supplementary concept word, rare disease supplementary concept word, unique identifier, synonyms, population supplementary concept word, anatomy supplementary concept word]  38. bangladesh.mp. [mp=title, book title, abstract, original title, name of substance word, subject heading word, floating sub-heading word, keyword heading word, organism supplementary concept word, protocol supplementary concept word, rare disease supplementary concept word, unique identifier, synonyms, population supplementary concept word, anatomy supplementary concept word]  39. sri lanka.mp. [mp=title, book title, abstract, original title, name of substance word, subject heading word, floating sub-heading word, keyword heading word, organism supplementary concept word, protocol supplementary concept word, rare disease supplementary concept word, unique identifier, synonyms, population supplementary concept word, anatomy supplementary concept word]  40. afghanistan.mp. [mp=title, book title, abstract, original title, name of substance word, subject heading word, floating sub-heading word, keyword heading word, organism supplementary concept word, protocol supplementary concept word, rare disease supplementary concept word, unique identifier, synonyms, population supplementary concept word, anatomy supplementary concept word]  41. 31 or 32 or 33 or 34 or 35 or 36 or 37 or 38 or 39 or 40  42. 30 and 41 |
| OVID MEDLINE (R) ALL 1946 to July 04, 2024 | 1. Afghanistan/  2. Afghanistan.ti,ab.  3. or/1-2 [LOW INCOME COUNTRIES IN SOUTH ASIA]  4. Bangladesh/  5. Bangladesh.ti,ab.  6. Bhutan/  7. Bhutan.ti,ab.  8. exp India/  9. India.ti,ab.  10. Nepal/  11. Nepal.ti,ab.  12. Pakistan/  13. Pakistan.ti,ab.  14. Sri Lanka/  15. (Sri Lanka or Ceylon).ti,ab.  16. or/4-15 [LOWER MIDDLE INCOME COUNTRIES IN SOUTH ASIA]  17. Maldives.ti,ab. [UPPER MIDDLE INCOME COUNTRIES IN SOUTH ASIA]  18. 1 or 2 or 3 or 4 or 5 or 6 or 7 or 8 or 9 or 10 or 11 or 12 or 13 or 14 or 15 or 16 or 17  19. exp Middle Aged/ or exp Female/ or exp Adolescent/ or exp Humans/  20. wom#n.mp. [mp=title, book title, abstract, original title, name of substance word, subject heading word, floating sub-heading word, keyword heading word, organism supplementary concept word, protocol supplementary concept word, rare disease supplementary concept word, unique identifier, synonyms, population supplementary concept word, anatomy supplementary concept word]  21. 19 or 20  22. 18 and 21  23. mental health.mp. [mp=title, book title, abstract, original title, name of substance word, subject heading word, floating sub-heading word, keyword heading word, organism supplementary concept word, protocol supplementary concept word, rare disease supplementary concept word, unique identifier, synonyms, population supplementary concept word, anatomy supplementary concept word]  24. exp Depression/cl, di, ec, pc, px [Classification, Diagnosis, Economics, Prevention & Control, Psychology]  25. exp Anxiety/ or exp Anxiety Disorders/  26. exp Financial Stress/ or exp Stress, Psychological/  27. exp Mental Health/ or exp "Quality of Life"/ or exp Depression/ or exp Stress, Psychological/  28. psychological well-being.mp. [mp=title, book title, abstract, original title, name of substance word, subject heading word, floating sub-heading word, keyword heading word, organism supplementary concept word, protocol supplementary concept word, rare disease supplementary concept word, unique identifier, synonyms, population supplementary concept word, anatomy supplementary concept word]  29. emotional well-being.mp. [mp=title, book title, abstract, original title, name of substance word, subject heading word, floating sub-heading word, keyword heading word, organism supplementary concept word, protocol supplementary concept word, rare disease supplementary concept word, unique identifier, synonyms, population supplementary concept word, anatomy supplementary concept word]  30. psychosocial stress.mp.  31. exp Depression/ or exp Mental Disorders/ or exp Anxiety Disorders/ or exp Depressive Disorder/  32. mental ill*.mp. [mp=title, book title, abstract, original title, name of substance word, subject heading word, floating sub-heading word, keyword heading word, organism supplementary concept word, protocol supplementary concept word, rare disease supplementary concept word, unique identifier, synonyms, population supplementary concept word, anatomy supplementary concept word]  33. 23 or 24 or 25 or 26 or 27 or 28 or 29 or 30 or 31 or 32  34. 22 and 33  35. economic intervention*.mp.  36. cash transfer*.mp. [mp=title, book title, abstract, original title, name of substance word, subject heading word, floating sub-heading word, keyword heading word, organism supplementary concept word, protocol supplementary concept word, rare disease supplementary concept word, unique identifier, synonyms, population supplementary concept word, anatomy supplementary concept word]  37. poverty alleviation.mp. [mp=title, book title, abstract, original title, name of substance word, subject heading word, floating sub-heading word, keyword heading word, organism supplementary concept word, protocol supplementary concept word, rare disease supplementary concept word, unique identifier, synonyms, population supplementary concept word, anatomy supplementary concept word]  38. poverty reduction.mp. [mp=title, book title, abstract, original title, name of substance word, subject heading word, floating sub-heading word, keyword heading word, organism supplementary concept word, protocol supplementary concept word, rare disease supplementary concept word, unique identifier, synonyms, population supplementary concept word, anatomy supplementary concept word]  39. social transfer.mp. [mp=title, book title, abstract, original title, name of substance word, subject heading word, floating sub-heading word, keyword heading word, organism supplementary concept word, protocol supplementary concept word, rare disease supplementary concept word, unique identifier, synonyms, population supplementary concept word, anatomy supplementary concept word]  40. social assistance.mp. [mp=title, book title, abstract, original title, name of substance word, subject heading word, floating sub-heading word, keyword heading word, organism supplementary concept word, protocol supplementary concept word, rare disease supplementary concept word, unique identifier, synonyms, population supplementary concept word, anatomy supplementary concept word]  41. income support.mp. [mp=title, book title, abstract, original title, name of substance word, subject heading word, floating sub-heading word, keyword heading word, organism supplementary concept word, protocol supplementary concept word, rare disease supplementary concept word, unique identifier, synonyms, population supplementary concept word, anatomy supplementary concept word]  42. livelihood*.mp. [mp=title, book title, abstract, original title, name of substance word, subject heading word, floating sub-heading word, keyword heading word, organism supplementary concept word, protocol supplementary concept word, rare disease supplementary concept word, unique identifier, synonyms, population supplementary concept word, anatomy supplementary concept word]  43. basic grant*.mp. [mp=title, book title, abstract, original title, name of substance word, subject heading word, floating sub-heading word, keyword heading word, organism supplementary concept word, protocol supplementary concept word, rare disease supplementary concept word, unique identifier, synonyms, population supplementary concept word, anatomy supplementary concept word]  44. insurance*.mp. [mp=title, book title, abstract, original title, name of substance word, subject heading word, floating sub-heading word, keyword heading word, organism supplementary concept word, protocol supplementary concept word, rare disease supplementary concept word, unique identifier, synonyms, population supplementary concept word, anatomy supplementary concept word]  45. loan*.mp. [mp=title, book title, abstract, original title, name of substance word, subject heading word, floating sub-heading word, keyword heading word, organism supplementary concept word, protocol supplementary concept word, rare disease supplementary concept word, unique identifier, synonyms, population supplementary concept word, anatomy supplementary concept word]  46. 35 or 36 or 37 or 38 or 39 or 40 or 41 or 42 or 43 or 44 or 45  47. 34 and 46 |
| Scopus (1979-2024) | ( ( ( ( TITLE-ABS-KEY ( female* ) OR TITLE-ABS-KEY ( woman ) OR TITLE-ABS-KEY ( gender ) ) ) AND ( ( TITLE-ABS-KEY ( mental AND health ) OR TITLE-ABS-KEY ( "mental disorder" ) OR TITLE-ABS-KEY ( "mental ill*" ) OR TITLE-ABS-KEY ( depression ) OR TITLE-ABS-KEY ( anxiety ) OR TITLE-ABS-KEY ( wellbeing ) OR TITLE-ABS-KEY ( stress* ) OR TITLE-ABS-KEY ( "psychosocial stress*" ) OR TITLE-ABS-KEY ( "common mental disorder*" ) ) ) ) AND ( ( TITLE-ABS-KEY ( "economic intervention" ) OR TITLE-ABS-KEY ( "cash transfer" ) OR TITLE-ABS-KEY ( poverty AND alleviation ) OR TITLE-ABS-KEY ( poverty AND reduction ) OR TITLE-ABS-KEY ( social AND transfer ) OR TITLE-ABS-KEY ( social AND assistance ) OR TITLE-ABS-KEY ( social AND welfare ) OR TITLE-ABS-KEY ( social AND income ) OR TITLE-ABS-KEY ( income AND support ) OR TITLE-ABS-KEY ( livelihood ) OR TITLE-ABS-KEY ( basic AND grant ) OR TITLE-ABS-KEY ( insurance ) OR TITLE-ABS-KEY ( loan ) ) ) ) AND ( ( TITLE-ABS-KEY ( "south asia" ) OR TITLE-ABS-KEY ( "global south" ) OR TITLE-ABS-KEY ( india ) OR TITLE-ABS-KEY ( maldives ) OR TITLE-ABS-KEY ( bangladesh ) OR TITLE-ABS-KEY ( nepal ) OR TITLE-ABS-KEY ( bhutan ) OR TITLE-ABS-KEY ( pakistan ) OR TITLE-ABS-KEY ( afghanistan ) ) ) AND ( LIMIT-TO ( SUBJAREA , "PSYC" ) OR LIMIT-TO ( SUBJAREA , "SOCI" ) OR LIMIT-TO ( SUBJAREA , "ECON" ) ) AND ( LIMIT-TO ( DOCTYPE , "ar" ) ) |
| 3ie Repository for Impact | ("Cash transfer" OR "poverty alleviation" OR "poverty reduction" OR "social transfer" OR "social assistance" OR "social welfare" OR "social income" OR "livelihood" OR "income support" OR "insurance" OR "loan" OR "basic grant" OR "Minimum income") AND ("mental disorder" OR "mental health" OR "mental illness" OR "well-being" OR "wellbeing" OR "depression" OR "stress" OR "anxiety" OR "depressive disorder" OR "anxiety disorder" OR "psychosocial stress" OR "common mental disorder") AND ("female" OR "women" OR "woman") AND ("South Asia" OR "Global South" OR "India" OR "Maldives" OR "Bangladesh" OR "Nepal" OR "Bhutan" OR "Pakistan" OR "Sri Lanka" OR "Afghanistan") |
| J-PAL | Hand searched |

Supplementary 3

**JBI Scoring method**

The general recommendation from JBI is to provide a narrative report of the synthesis of the critical appraisal (Barker *et al.*, 2024). To facilitate this report, I devised a points-based system to assess the risk of bias for all included studies. Each response was assigned points: yes (+2), unclear/maybe (+1), no (0). The maximum points for each critical appraisal tool were calculated by multiplying the total number of questions by 2 as a ‘yes’ response gives the maximum points. The cut-offs for total risk of bias were based on percentage: low (75% to 100%), moderate (50% to 74%), high (<50%). Table 2 provides total risk of bias cut-offs for each study design.

**Table: Total risk of bias cut-offs for JBI checklist**

| JBI tool | Maximum points | Total risk of bias cut-offs | | |
| --- | --- | --- | --- | --- |
|  |  | **Low (75%-100%)** | **Moderate (74%-50%)** | **High (<50%)** |
| Cross-sectional | 16 | 12-16 | 8-11 | <8 |
| Quasi-experimental | 18 | 14-18 | 9-13 | <9 |
| RCT | 26 | 20-26 | 13-19 | <13 |

**Cross-Sectional Studies**

|  | **Scott et al., 2020** | **Kayser et al., 2010** | **Newransky, Kayser & Lombe, 2014** | **Yount et al., 2021** |
| --- | --- | --- | --- | --- |
| Were the criteria for inclusion in the sample clearly defined? | 2 | 2 | 2 | 2 |
| Were the study subjects and the setting described in detail | 2 | 2 | 2 | 2 |
| Was the exposure measured in a valid and reliable way | 1 | 2 | 2 | 2 |
| Were objective, standard criteria used for measurement of the condition | 2 | 0 | 0 | 2 |
| Were confounding factors identified | 1 | 1 | 1 | 1 |
| Were strategies to deal with confounding factors stated | 1 | 1 | 1 | 1 |
| Were the outcomes measured in a valid and reliable way | 2 | 1 | 1 | 2 |
| Was appropriate statistical analysis used | 2 | 2 | 2 | 2 |
| Total Risk of Bias | 13  **Low** | 11  **Moderate** | 11  **Moderate** | 14  **Low** |

**RCT**

|  | **Gibbs et al., 2020** |
| --- | --- |
| Was true randomization used for assignment of participants to treatment groups | 2 |
| Was allocation to treatment groups concealed | 2 |
| Were treatment groups similar at the baseline | 2 |
| Were participants blind to treatment assignment | 0 |
| Were those delivering the treatment blind to treatment assignment | 0 |
| Were treatment groups treated identically other than the intervention of interest? | 2 |
| Were outcome assessors blind to treatment assignment? | 0 |
| Were outcomes measured in the same way for treatment groups? | 2 |
| Were outcomes measured in a reliable way | 2 |
| Was follow up complete and if not, were differences between groups in terms of their follow up adequately described and analysed? | 2 |
| Were participants analysed in the groups to which they were randomized? | 2 |
| Was appropriate statistical analysis used? | 2 |
| Was the trial design appropriate and any deviations from the standard RCT design (individual randomization, parallel groups) accounted for in the conduct and analysis of the trial? | 2 |
| Total Risk of Bias | 20  **Low** |

**Quasi Experimental Studies**

|  | **Tsaneva & Balakrishnan, 2019** | **Jalal, Frongillo & Warren, 2015** | **Powell-Jackson et al., 2016** |
| --- | --- | --- | --- |
| Is it clear in the study what is the “cause” and what is the “effect” (i.e. there is no confusion about which variable comes first)? | 2 | 2 | 2 |
| Was there a control group | 2 | 2 | 2 |
| Were participants included in any comparisons similar | 0 | 0 | 2 |
| Were the participants included in any comparisons receiving similar treatment/care, other than the exposure or intervention of interest? | 2 | 2 | 2 |
| Were there multiple measurements of the outcome, both pre and post the intervention/exposure | 0 | 0 | 0 |
| Were the outcomes of participants included in any comparisons measured in the same way? | 2 | 2 | 2 |
| Were outcomes measured in a reliable way? | 1 | 2 | 1 |
| Was follow-up complete and if not, were differences between groups in terms of their follow-up adequately described and analyzed | 0 | 0 | 0 |
| Was appropriate statistical analysis used? | 2 | 2 | 2 |
| Total Risk of Bias | 11  **Moderate** | 12  **Moderate** | 13  **Moderate** |
